# In vitro metabolism of synthetic cannabinoid AM1220 by human liver microsomes and *Cunninghamella elegans* using liquid chromatography coupled with high resolution mass spectrometry

**DOI:** 10.1007/s11419-018-0424-y

**Published:** 2018-05-24

**Authors:** Shimpei Watanabe, Unnikrishnan Kuzhiumparambil, Shanlin Fu

**Affiliations:** 1 Centre for Forensic Science, School of Mathematical and Physical Sciences, University of Technology Sydney (UTS), PO Box 123, Broadway, NSW 2007 Australia; 2 Climate Change Cluster, University of Technology Sydney (UTS), PO Box 123, Broadway, NSW 2007 Australia

**Keywords:** AM1220, Synthetic cannabinoid, In vitro metabolism, Human liver microsomes, *Cunninghamella elegans*, High resolution mass spectrometry

## Abstract

**Purpose:**

Identifying intake of synthetic cannabinoids generally requires the metabolism data of the drugs so that appropriate metabolite markers can be targeted in urine testing. However, the continuous appearance of new cannabinoids during the last decade has made it difficult to keep up with all the compounds including {1-[(1-methylpiperidin-2-yl)methyl]-1*H*-indol-3-yl}(naphthalen-1-yl)methanone (AM1220). In this study, metabolism of AM1220 was investigated with human liver microsomes and the fungus *Cunninghamella elegans*.

**Methods:**

Metabolic stability of AM1220 was analysed by liquid chromatography–tandem mass spectrometry in multiple reaction monitoring mode after 1 µM incubation in human liver microsomes for 30 min. Tentative structure elucidation of metabolites was performed on both human liver microsome and fungal incubation samples using liquid chromatography–high-resolution mass spectrometry.

**Results:**

Half-life of AM1220 was estimated to be 3.7 min, indicating a high clearance drug. Nine metabolites were detected after incubating human liver microsomes while seven were found after incubating *Cunninghamella elegans*, leading to 11 metabolites in total (five metabolites were common to both systems). Demethylation, dihydrodiol formation, combination of the two, hydroxylation and dihydroxylation were the observed biotransformations.

**Conclusions:**

Three most abundant metabolites in both human liver microsomes and *Cunninghamella elegans* were desmethyl, dihydrodiol and hydroxy metabolites, despite different isomers of dihydrodiol and hydroxy metabolites in each model. These abundant metabolites can potentially be useful markers in urinalysis for AM1220 intake.

## Introduction

AM1220, or {1-[(1-methylpiperidin-2-yl)methyl]-1*H*-indol-3-yl}(naphthalen-1-yl)methanone, is a synthetic cannabinoid that was first synthesised in the 1990s to study the structure-activity relationship of cannabinoid receptors [1]. The cannabinoid was shown to have a binding affinity (*K*_i_) of 3.88 and 73.4 nM to cannabinoid receptor type 1 (CB_1_) and type 2 (CB_2_) receptors, respectively [2]. Because of the high affinity to cannabinoid receptors, AM1220 began to be sold and abused as “herbal products” and “research chemicals” on the recreational drug market among the continuous emergence of a myriad of new psychoactive substances [3–8].

In these products, AM1220 is usually found together with its azepane isomer, [1-(1-methyl-3-azepanyl)-1*H*-indol-3-yl](1-naphthyl)methanone [4, 5], which is suggested to be present as a synthetic impurity [4] or due to a rearrangement that occurs over time [7]. The presence of the AM1220 azepane isomer may complicate interpretation of the pharmacological effects of AM1220, as the azepane isomer itself is shown to have binding affinities to CB_1_ and CB_2_ receptors [9].

For detection of synthetic cannabinoids in humans, plasma samples are shown to be useful since the parent drugs can be found as they are without modifications [10]. However, there are some issues with detection in plasma samples. Firstly, the window of detection of the parent drugs in blood is short [10, 11]. Secondly, the concentrations of the parent drugs in plasma are reported to be lower than those of the major metabolites [11]. In addition, plasma samples are not always obtainable due to invasiveness of collection method, and urine samples are often the preferred choice for drug testing. Therefore, suitable methods to analyse urine samples are desirable. Nevertheless, synthetic cannabinoids are highly lipophilic, and high distribution rate of parent drugs for tissue such as fat results in low excretion rate in urine. Furthermore, synthetic cannabinoids are extensively metabolised in humans and are generally not excreted in urine in the parent drug form. Consequently, metabolites need to be monitored for detecting synthetic cannabinoids in urine specimens.

Metabolism studies of synthetic cannabinoids have been performed using several approaches. Human liver microsome (HLM) incubation is the most common in vitro approach, and even though not reflective of the metabolism in a whole human body, it can generate a wide variety of human metabolites with advantages such as low cost and larger pools of donors [11–13]. Human hepatocytes provide the metabolic profiles closest to the in vivo human data [14–16], and animal models such as rats are valuable as a source of in vivo data, though not always consistent with human findings [17–19]. Incubation with the fungus *Cunninghamella elegans* (*C. elegans*) has been shown to produce similar metabolic profiles to the human system with the advantage of low cost and production of large quantity of metabolites [20–22]. *Cunninghamella elegans* is, however, not suitable for strict absorption-distribution-metabolism-elimination (ADME) studies, since it does not provide blood and urine as separate specimens as animal models do. The presence and abundance of the metabolites determined by these models may not be an accurate representation of in vivo metabolites. Thus, the in vitro metabolites should be confirmed in human urine, if available, by analysis of urine samples obtained from suspected users of synthetic cannabinoids, since analysis of human urine from controlled administration is difficult at this point without sufficient data to ensure safety [11].

To date, there has been no in vitro metabolism study of AM1220. There is one in vivo study by Zaitsu et al. [10] reporting two metabolites of AM1220 and two more potential metabolites in postmortem human plasma and urine specimens, respectively, from a fatal intoxication case. To complement the in vivo findings, which may have been affected by genotype, phenotype and/or inhibition of cytochrome P450 (CYP) enzymes by coadministration of drug, in vitro metabolism study will be useful [23].

In this study, we report the metabolic stability of AM1220 based on HLM incubation and tentative structure elucidation of AM1220 metabolites obtained from HLM and *C. elegans* incubation. Suitable markers for urinalysis are also suggested. Liquid chromatography–quadrupole time-of-flight mass spectrometry (LC–QTOF-MS) was used for analysis since high-resolution mass spectrometry has an advantage of providing accurate masses, enabling more confident characterisation of metabolites [24].

## Materials and methods

### Chemicals and reagents

AM1220 was obtained from Cayman Chemical (Ann Arbor, MI, USA). UR-144 was synthesised in-house following the methods previously reported [25, 26] and characterised by mass spectrometry (MS) and 1D and 2D nuclear magnetic resonance spectroscopy techniques. Fifty-donor HLM pool, NADPH system solution A and NADPH system solution B were from Corning (Corning, NY, USA). Liquid chromatography–mass spectrometry (LC–MS) grade acetonitrile was obtained from Honeywell (Muskegon, MI, USA). Reagent grade dichloromethane and sodium chloride were purchased from Chemsupply (Gilman, SA, Australia). LC–MS grade formic acid was obtained from Sigma-Aldrich (St. Louis, MO, USA). *Cunninghamella elegans* ATCC 10028b was from Cryosite Ltd. (South Granville, NSW, Australia). Glycerol and potassium dihydrogen phosphate and dipotassium hydrogen phosphate were from Ajax Chemicals (Auburn, NSW, Australia). Potato dextrose agar, glucose, peptone, and yeast extract were purchased from Oxoid Australia (Adelaide, SA, Australia).

### Metabolic stability

AM1220 solution in acetonitrile/phosphate buffer (40 µM, pH 7.4, 25 µL, 0.1% acetonitrile), phosphate buffer (0.1 M, pH 7.4, 855 µL), NADPH-A (50 µL) and NADPH-B (20 µL) were mixed in an Eppendorf tube, to which HLM (50 µL = 1 mg protein) was added. The final concentration of AM1220 in the mixture was 1 µM with 0.003% acetonitrile. The mixture was incubated in triplicate at 37 °C in a shaking water bath. At time 0, 3, 8, 13, 20 and 30 min, a 100-µL aliquot was removed and placed into 100 µL ice-cold acetonitrile to quench the reaction. The mixture was centrifuged at 16,060 × *g* for 10 min and filtered with a 0.22 µm filter. Ten microliters of the filtrate was diluted in 990 µL water/acetonitrile (70:30, v/v) and 10 µL was injected into liquid chromatography–triple quadrupole mass spectrometer in triplicate.

Chromatographic separation was performed on an Agilent 1290 LC system with an Agilent Zorbax Eclipse XDBC18 analytical column (150 × 4.6 mm i.d., particle size 5 μm) (Agilent Technologies, Santa Clara, CA, USA). The mobile phase consisted of 0.1% formic acid in water (A) and 0.1% formic acid in acetonitrile (B). The gradient system was as follows: 30% B until 1 min, ramped to 40% B over 15 min, 95% B at 16.01 min and held until 19.1 min, ramped down to 30% B at 19.11 min and held until 23 min. The flow rate was 0.4 mL/min and the column temperature was kept at 30 °C.

Mass spectrometry was run in multiple reaction monitoring mode on an Agilent 6490 Triple Quadrupole mass spectrometer with electrospray ionisation (ESI) source in positive ion mode (Agilent Technologies). Two transitions (*m/z* 383 → 286 and *m/z* 383 → 98) were monitored with fragmentor voltage of 380 V and collision energy of 20 and 50 eV, respectively.

In vitro microsomal half-life (*t*_1/2_) of AM1220 was calculated based on the plot of natural log of percentage of the drug remaining against time. Percentage of the drug remaining was calculated by dividing the peak area of the drug remaining at each time point by the peak area of the drug at time 0 min and multiplying by 100%. The slope of the line (−*k*) was used to give *t*_1/2_ = ln2/k. Intrinsic clearance (CL_int_, in mL/min/kg) was calculated based on the following formula [27]:

$${\text{CL}}_{\text{int}} = \frac{\ln 2}{{t_{1/2} }} \times \frac{\text{mL of incubation}}{\text{mg of microsomes}} \times \frac{{45{\text{ mg of microsomes}}}}{\text{g of liver}} \times \frac{{ 2 0 {\text{ g of liver}}}}{\text{kg of body weight}},$$

where *t*_1/2_ (the only variable in the equation) was substituted.

Hepatic clearance (CL_H_) and hepatic extraction ratio (*E*_H_) were calculated based on the well-stirred model from the following formulae without considering blood protein and microsome binding [27, 28]. The 21 mL/min/kg was used for human hepatic blood flow (*Q*_H_) [27].

$${\text{CL}}_{\text{H}} = \frac{{Q_{\text{H}} \times {\text{CL}}_{\text{int}} }}{{Q_{\text{H}} + {\text{CL}}_{\text{int}} }},$$

$$E_{\text{H}} = \frac{{{\text{CL}}_{\text{H}} }}{{Q_{\text{H}} }}.$$

### Tentative structure elucidation of metabolites

#### Human liver microsome incubation

The incubation mixture was prepared as described for the metabolic stability study using 1 mg/mL, i.e., 2.61 mM AM1220 solution (final concentration of acetonitrile was 0.2%). The mixture was incubated at 37 °C in a shaking water bath for 1 h. The reaction was quenched by adding ice-cold acetonitrile (1 mL) to the mixture and it was centrifuged at 16,060 × *g* for 10 min. The sample was filtered (0.22 µm) and injected to LC–QTOF-MS. A control sample without HLM, a control without AM1220 and a positive control using UR-144 were also incubated and analysed.

#### Fungus incubation

*Cunninghamella elegans* was cultured on potato dextrose agar plates at 27 °C for 5 days. The mycelia of the fungus were mixed in sterile physiological saline solution (1 plate of mycelia/5 mL). Growth medium was prepared [29], and 1.5 mL of the fungus solution was added to 100 mL of medium in a conical flask. The culture was incubated for 48 h at 26 °C and 180 rpm on an Infors HT Multitron rotary shaker (In Vitro Technologies, Noble Park North, VIC, Australia). AM1220 (1 mg in 0.5 mL acetonitrile) was added to the flask and incubated for another 72 h. The solution was filtered, extracted with dichloromethane (3 × 50 mL) and evaporated using a rotary evaporator and a vacuum pump. The sample was reconstituted in 2 mL acetonitrile, which was further diluted in acetonitrile tenfold. A control without fungus and a control without AM1220 were also incubated.

#### LC–QTOF-MS

Chromatographic equipment and conditions were the same as described above for metabolic stability section, except for the following. The gradient started with 30% B, and was held until 1 min, ramped up to 40% B over 19 min, 90% B at 21 min, held until 24 min, ramped down to 30% B at 25 min and held until 30 min for re-equilibration. Injection volume was 2 µL for scan analysis and 10 µL for product ion scan analysis.

Mass spectra were acquired on an Agilent 6510 Accurate Mass Q-TOF mass spectrometer, equipped with a dual ESI source (Agilent Technologies). The parameters were as follows: scanning mass range, *m/z* 100–1000 (MS), *m/z* 80–1000 (MS/MS); capillary voltage, 3500 V; nebulizer pressure, 30 psig; gas temperature, 325 °C; gas flow, 5 L/min; fragmentor voltage, 160 V; collision energy for product ion scan analysis, 10, 20 and 40 eV; skimmer voltage, 65 V. Mass calibration was performed with the mixture provided by the manufacturer. Real-time mass calibration was enabled using the following reference masses: *m/z* 121.0509 and 922.0098.

Additional MS analyses were performed on an Agilent 6550A iFunnel Q-TOF with a dual AJS ESI source (Agilent Technologies) operated with the same parameters as above except for the following: gas temperature, 290 °C; gas flow, 11 L/min; sheath gas temperature, 350 °C; sheath gas flow, 11 L/min; injection volume for product ion scan analysis, 2 µL.

Extracted ion chromatograms and mass spectra were analysed using Agilent MassHunter Workstation Software Qualitative Analysis (version B.06.00). A personal compound database and library (PCDL) with known and potential metabolites of the drug was created with Agilent MassHunter PCDL Manager (version B.04.00) to search for the metabolites. Search parameters were as follows: mass tolerance, 20 ppm; maximum number of matches, 8; absolute peak area ≥ 5000. The criteria for metabolites were as follows: mass error of the protonated molecules ≤ 5 ppm; consistent fragmentation pattern with proposed structure; reasonable retention time relative to other biotransformations; absence of the metabolite in controls.

## Results

### Metabolic stability

In vitro *t*_1/2_ of AM1220 was calculated to be 3.7 ± 0.4 min [mean ± standard deviation (SD), *n* = 3]. From the calculated *t*_1/2_ value, CL_int_, CL_H_ and *E*_H_ were estimated to be 168.5 mL/min/kg, 18.7 mL/min/kg, and 0.89, respectively. The percentage of drug remaining at each time point, used for calculation, is shown in Table 1.

**Table 1:** Average percentage of the drug remaining and relative standard deviation (RSD, *n* = 3) at each time point for three human liver microsome (HLM) incubation samples for metabolic stability study

	Time (min)	Average percentage of the drug remaining (%)	RSD (%)
Sample 1	0	100	0
	3	44.4	0.9
	8	14.9	0.6
	13	4.6	2.1
	20	1.0	7.0
	30	0.2	14.7
Sample 2	0	100	0
	3	46.9	0.3
	8	14.9	1.0
	13	5.3	1.1
	20	1.0	3.1
	30	0.3	30.2
Sample 3	0	100	0
	3	55.9	1.5
	8	18.9	0.2
	13	6.7	0.6
	20	2.0	4.2
	30	0.8	4.6

### Tentative structure elucidation of metabolites

**Fig. 1:**
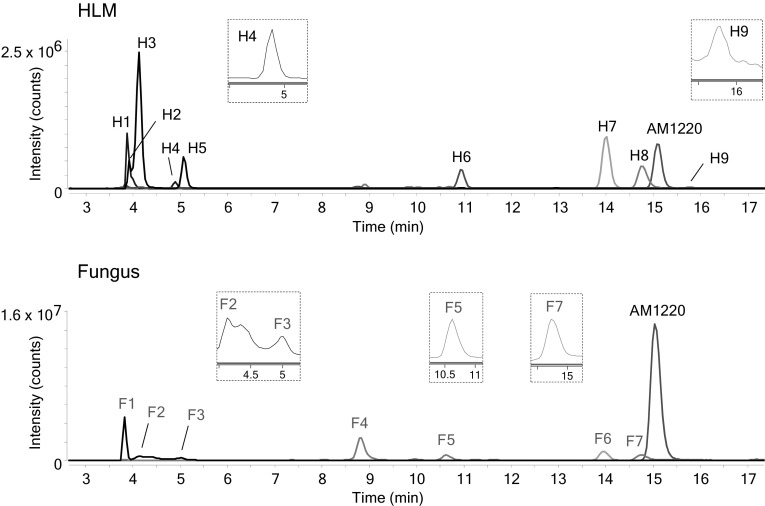
Combined extracted ion chromatograms, from total ion current chromatogram, of AM1220 and its metabolites in human liver microsome (HLM) and fungus incubation

**Fig. 2:**
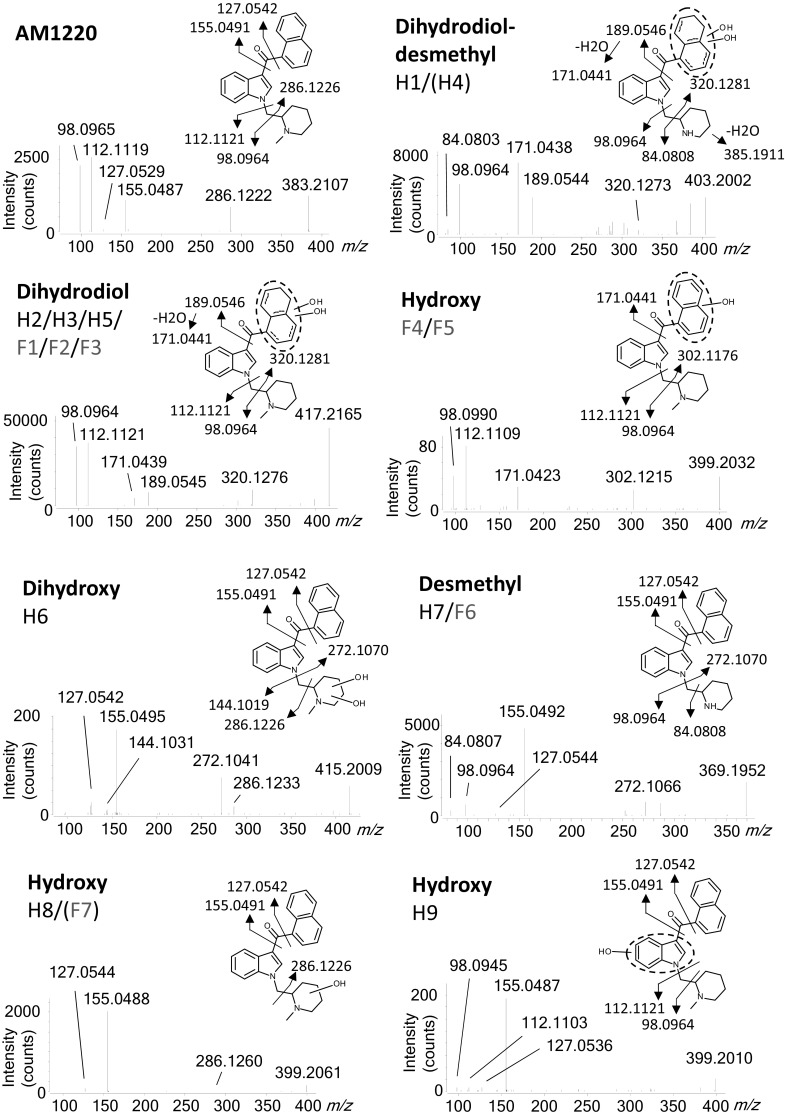
Product ion spectra of AM1220 and its metabolites at collision energy of 20 eV, and proposed metabolite structures with exact masses of fragmentation. Metabolites in brackets did not show all the product ions. The exact locations of dihydrodiol groups in (F1–F3 and H1–H5) were not determined

**Fig. 3:**
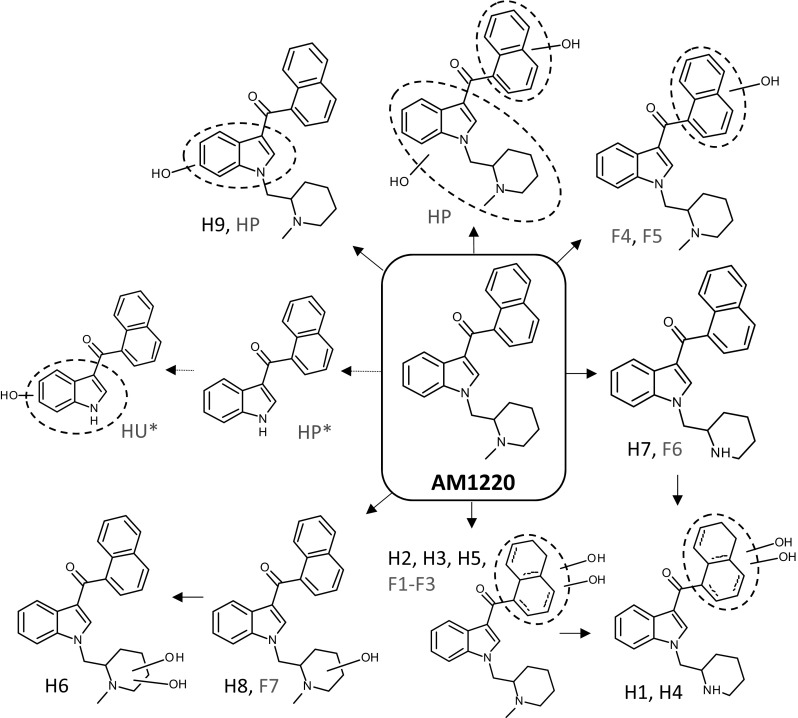
Proposed metabolic pathway of AM1220 in HLM and fungus *C. elegans* incubation in comparison with postmortem human data from the literature [10]. HP and HU refer to human plasma and human urine samples, while asterisk indicates that the origin of the metabolite is not confirmed to be AM1220. The exact positions of hydroxy groups in F4/F5 and H9 and dihydrodiol groups in F1–F3 and H1–H5 were not determined

Chromatograms of AM1220 and its metabolites after HLM and *C. elegans* incubation are shown in Fig. 1. The product ion spectra and the suggested fragmentation patterns of AM1220 and the metabolites are shown in Fig. 2. The proposed metabolic pathway of AM1220 in HLM and *C. elegans* incubation is compared with the in vivo postmortem human data in the literature (Fig. 3). Table 2 lists all the metabolites with retention times, elemental compositions, exact mass, accurate mass, mass errors, diagnostic product ions and chromatographic peak areas.

**Table 2:** AM1220 metabolites after HLM and fungus *C. elegans* incubation

ID		Metabolites	RT (min)	Elemental composition [M + H]	Exact mass	Accurate mass		Mass error (ppm)		Diagnostic product ions	Area	
HLM	Fungi					HLM	Fungi	HLM	Fungi		HLM	Fungi
H1		Dihydrodiol formation + demethylation	3.9	C25H27N2O3	403.2016	403.2015		0.17		84, 98, 171, 189, 320	5.55E + 06	
				C20H18NO3	320.1281	320.1273		− 2.50				
				C11H9O3	189.0546	189.0544		− 1.06				
				C11H7O2	171.0441	171.0438		− 1.75				
				C6H12 N	98.0964	98.0964		0				
				C5H10 N	84.0808	84.0803		− 5.95				
H2	F1	Dihydrodiol formation	3.9	C26H29N2O3	417.2173	417.2173	417.2168	0.16	1.15	98, 112, 171, 189, 320	7.84E + 05	2.54E + 07
				C20H18NO3	320.1281	320.1275	320.1276	− 1.87	− 1.56			
				C11H9O3	189.0546	189.0542	189.0545	− 2.12	− 0.53			
				C11H7O2	171.0441	171.0434	171.0439	− 4.09	− 1.17			
				C7H14 N	112.1121	112.1121	112.1122	0	0.89			
				C6H12 N	98.0964	98.0963	98.0966	− 1.02	2.04			
H3	F2	Dihydrodiol formation	4.1	C26H29N2O3	417.2173	417.2168	417.2171	− 1.15	− 0.84	98, 112, 171, 189, 320	1.93E + 07	4.56E + 06
				C20H18NO3	320.1281	320.1276	320.1275	− 1.56	− 1.87			
				C11H9O3	189.0546	189.0545	189.0549	− 0.53	1.59			
				C11H7O2	171.0441	171.0439	171.0439	− 1.17	− 1.17			
				C7H14 N	112.1121	112.1121	112.1122	0	0.89			
				C6H12 N	98.0964	98.0964	98.0965	0	1.02			
H4		Dihydrodiol formation + demethylation	4.9	C25H27N2O3	403.2016	403.2012		− 0.83		84, 98, 171, 385	9.72E + 05	
				C25H25N2O2	385.1911	385.1902		− 2.34				
				C11H7O2	171.0441	171.0444		1.75				
				C6H12 N	98.0964	98.0963		− 1.02				
				C5H10 N	84.0808	84.0809		1.19				
H5	F3	Dihydrodiol formation	5.1	C26H29N2O3	417.2173	417.2168	417.2158	− 1.06	1.08	98, 112, 171, 189, 320	4.41E + 06	4.99E + 06
				C20H18NO3	320.1281	320.1270	320.1271	− 3.44	− 3.12			
				C11H9O3	189.0546	189.0540	189.0546	− 3.17	0			
				C11H7O2	171.0441	171.0436	171.0436	− 2.92	− 2.92			
				C7H14 N	112.1121	112.1121	112.1121	0	0			
				C6H12 N	98.0964	98.0964	98.0965	0	1.02			
	F4	Hydroxylation	8.8	C26H27N2O2	399.2067		399.2066		− 0.34	98, 112, 171, 302		3.43E + 07
				C20H16NO2	302.1176		302.1215		12.91			
				C11H7O2	171.0441		171.0423		− 10.52			
				C7H14 N	112.1121		112.1109		− 10.70			
				C6H12 N	98.0964		98.0990		26.50			
	F5	Hydroxylation	10.6	C26H27N2O2	399.2067		399.2067		− 0.83	98, 112, 171, 302		8.07E + 06
				C20H16NO2	302.1176		302.1158		− 5.96			
				C11H7O2	171.0441		171.0447		3.51			
				C7H14 N	112.1121		112.1102		− 16.95			
				C6H12 N	98.0964		98.0980		16.31			
H6		Dihydroxylation	10.9	C26H27N2O3	415.2016	415.2010		0.72		127, 144, 155, 272, 286	3.65E + 06	
				C20H16NO	286.1226	286.1233		2.45				
				C19H14NO	272.1070	272.1041		− 10.66				
				C11H7O	155.0491	155.0495		2.58				
				C7H14NO2	144.1019	144.1031		8.33				
				C10H7	127.0542	127.0542		0				
H7	F6	Demethylation	14.0	C25H25N2O	369.1961	369.1962	369.1961	0.25	0.16	84, 98, 127, 155, 272	1.19E + 07	1.36E + 07
				C19H14NO	272.1070	272.1066	272.1063	− 1.47	− 2.57			
				C11H7O	155.0491	155.0492	155.0488	0.64	− 1.93			
				C10H7	127.0542	127.0544	127.0533	1.57	− 7.08			
				C6H12 N	98.0964	98.0964	98.0964	0	0			
				C5H10 N	84.0808	84.0807	84.0807	− 1.19	− 1.19			
H8	F7	Hydroxylation	14.8	C26H27N2O2	399.2067	399.2061	399.2067	0.27	0.35	127, 155, 286^a^	6.23E + 06	1.14E + 07
				C20H16NO	286.1226	286.1260		11.88				
				C11H7O	155.0491	155.0488	155.0490	− 1.93	-0.64			
				C10H7	127.0542	127.0544	127.0547	1.57	3.94			
Parent		AM1220	15.1	C26H27N2O	383.2118	383.2118	383.2116	0.62	− 0.22	98, 112, 127, 155, 286	1.03E + 07	2.21E + 08
				C20H16NO	286.1226	286.1222	286.1222	− 1.40	− 1.40			
				C11H7O	155.0491	155.0487	155.0487	− 2.58	− 2.58			
				C10H7	127.0542	127.0529	127.0538	− 10.23	− 3.15			
				C7H14 N	112.1121	112.1119	112.1121	− 1.78	0			
				C6H12 N	98.0964	98.0965	98.0965	1.02	1.02			
H9		Hydroxylation	15.8	C26H27N2O2	399.2067	399.2058		− 2.17		98, 112, 127, 155	5.57E + 05	
				C11H7O	155.0491	155.0487		− 2.58				
				C10H7	127.0542	127.0536		− 4.72				
				C7H14 N	112.1121	112.1103		− 16.06				
				C6H12 N	98.0964	98.0945		− 19.37				

*RT* retention time

^a^Not found in F7

#### Human liver microsomes

Nine metabolites were detected in HLM incubation and assigned as H1–H9 in the order of retention time (Fig. 1). The following metabolites were detected; dihydrodiol (H2, H3, H5), dihydrodiol with demethylation (H1, H4), demethylation (H7), hydroxylation (H8, H9) and dihydroxylation (H6). The mass errors of the metabolites compared with the proposed elemental compositions were ≤ 2.17 ppm (Table 2). The top three abundant metabolites based on the peak area were dihydrodiol (H3), demethylation (H7) and hydroxylation (H8) metabolites.

#### Fungus *C. elegans*

Seven metabolites were found after *C. elegans* incubation and assigned as F1–F7 (Fig. 1). Dihydrodiol (F1–F3), demethylation (F6) and hydroxylation (F4, F5, F7) metabolites were identified. Five of them were the same metabolites as HLM metabolites (Table 2); dihydrodiol (H2 and F1, H3 and F2, H5 and F3), demethylation (H7 and F6) and hydroxylation (H8 and F7) were common metabolites between HLM and fungus metabolism. The mass errors were all ≤ 1.15 ppm. The three most abundant metabolites were hydroxylation (F4), dihydrodiol (F1) and demethylation (F6) products.

## Discussion

### Metabolic stability

In vitro *t*_1/2_ of AM1220 was 3.7 min and this belongs to the class of high clearance compounds [30]. The estimated *E*_H_ of 0.89 also indicates high extraction, suggesting the compound to be highly susceptible to hepatic metabolism [31]. These findings are in line with other synthetic cannabinoids and account for the extensive metabolism of cannabinoids [15, 32, 33].

### Tentative structure elucidation of metabolites

Nine and seven metabolites were detected after incubation of AM1220 with HLM and fungus, respectively. Based on the retention times and the fragmentation patterns of the metabolites, five of them were considered identical and hence a total of 11 metabolites were found from two in vitro models (Table 2). The tentative structure elucidation of these metabolites is described below.

#### Hydroxylation

Four hydroxylated metabolites were detected at *m/z* 399. F4 and F5 showed product ions at *m/z* 171 and 302, which were 16 amu higher than the unchanged naphthoyl moiety (*m/z* 155) and 1-methyl-3-naphthoylindole *(m/z* 286), respectively, indicating hydroxylation at the naphthalene moiety. Other product ions at *m/z* 98 and 112 confirmed the piperidine moiety to be unaltered. H8 was shown to be hydroxylated at the methylpiperidine moiety by the intact product ions at *m/z* 127, 155, and 286, indicative of the unmodified indole and naphthalene moieties. The absence of ions at *m/z* 98 and 112 also indicated the modification of the methylpiperidine ring. F7 eluted at the same retention time as H8, but only showed the product ions at *m/z* 127 and 155 without 286. Without the ion at *m/z* 286, hydroxylation could have occurred at either indole or piperidine moiety. However, it was considered to be the identical metabolite as H8 because of the same retention time and the fact that the ions at *m/z* 98 and 112 were absent, which were seen for all the other metabolites without modification to the piperidine moiety and the parent drug. H9 was characterised by the unchanged naphthalene (*m/z* 127, 155) and unchanged piperidine (*m/z* 98, 112), indicating the location of hydroxylation to be the indole ring.

#### Dihydroxylation

A dihydroxy metabolite (H6) was found at *m/z* 415, which resulted from further oxidation of H8. The fragment ions at *m/z* 127, 155 and 286 are in common with the parent drug, indicating the intact naphthoylindole moiety. The ion at *m/z* 144 indicated the (1-methyl-2-piperidinyl)methyl moiety to be the site of dihydroxylation. It is interesting to note that an abundant fragment ion at *m/z* 272 was observed. While the presence of the ion does not contradict the aforementioned position of dihydroxylation, this ion was formed from a different fragmentation pattern from the parent drug.

#### Dihydrodiol formation

Three dihydrodiol metabolites (H2/F1, H3/F2 and H5/F3) were observed with *m/z* 417. All three metabolites showed the same fragment ions: *m/z* 98, 112, 171, 189 and 320. The ions at *m/z* 98 and 112 show the unchanged piperidine moiety while *m/z* 189 and 320 indicate dihydrodiol formation at naphthalene moiety with the former losing a water molecule to form *m/z* 171.

#### Demethylation

A metabolite demethylated at the piperidine nitrogen (H7/F6) was detected at *m/z* 369. The product ions at *m/z* 127 and 155 were retained as the naphthalene moiety is intact. The fragment ions at *m/z* 98 and 272 were generated by *N*-dealkylation of indole, corresponding to a demethylated piperidine moiety and the unaltered naphthoylindole, respectively (Fig. 2). The product ion at *m/z* 98 further lost a methylene moiety to form the ion at *m/z* 84. The lack of product ion at *m/z* 112 also supports demethylation of the methylpiperidine moiety.

#### Dihydrodiol formation and demethylation

Two metabolites at *m/z* 403 (H1, H4) were found to have undergone both dihydrodiol formation at the naphthalene ring and demethylation of the methylpiperidine moiety. For H1, the combination of the product ions at *m/z* 171, 189 and 320 indicates the formation of dihydrodiol at the naphthalene moiety, whereas the ions at *m/z* 84 and 98 without 112 reflect a demethylated piperidine ring. In fact, H4 did not show the fragment ion at *m/z* 189, but this is probably because the dihydrodiol at a particular position is less stable and easily loses a water molecule [34]. The hypothesis is supported by the observation that the fragment ion at *m/z* 385, resulting from water loss of the molecule, is prominent in H4.

### Comparison of AM1220 metabolites in HLM and *C. elegans* with in vivo human metabolites

Out of nine HLM and seven fungal metabolites detected in this study, five metabolites were found to be identical, i.e., more than 50% of HLM and fungal metabolites were the same as each other. In terms of the biotransformations of AM1220, dihydrodiol formation, demethylation and hydroxylation were the common transformations between HLM and fungal metabolites. HLM additionally showed the transformations by dihydroxylation and combinations of dihydrodiol formation and demethylation. Overall, metabolism of AM1220 by HLM and *C. elegans* was highly consistent.

*Cunninghamella elegans* is known to contain CYP509A1, closely related to the CYP51 family [35], and it can perform a number of reactions including both phase I and phase II biotransformations [36]. Enzymes responsible for hydroxylation, *N*-demethylation, sulfation, glucuronidation, glycosylation, and glutathione conjugation have been indicated to be present [37], and recently, the presence of CYP3A4 in *C. elegans* was indicated [38]. In addition, the fungus has facilitated some biotransformations, which were catalysed by CYP1A2, CYP2C9, CYP2C19 and CYP2D6 in human metabolism [20, 39–43]. Although the enzymes responsible for the metabolic transformations of AM1220 in HLM or *C. elegans* are unknown, the presence of similar enzymes in both models is likely the factor for their high consistency.

To date, the study by Zaitsu et al. [10] is the only one reporting the in vivo human metabolites of AM1220. In the study, four metabolites were detected from a fatal case of intoxication; hydroxylation, dihydroxylation and *N*-dealkylation in plasma and *N*-dealkylation followed by hydroxylation in urine (Fig. 3). It should be noted, however, that metabolites of *N*-dealkylation and *N*-dealkylation followed by hydroxylation were not confirmed to have formed from AM1220 because AM-2232, which also contains a naphthoylindole moiety and hence another potential source of these metabolites, was also detected in plasma.

Out of the four metabolites, *N*-dealkylation and *N*-dealkylation followed by hydroxylation were not observed after either HLM or *C. elegans* incubation. Dihydroxylation was detected in HLM incubation (H6), yet the positions of hydroxy groups were different; dihydroxylation took place at the piperidine moiety in H6, while one hydroxylation at the naphthalene moiety and another at either the indole or piperidine moiety in the plasma metabolite. The hydroxylated metabolite is the only metabolite potentially in common with HLM incubation, as it may be identical to H9 based on the mass fragmentation pattern. The inconsistency between the in vitro and in vivo metabolites may be due to the genotype/phenotype and/or the coadministration of CYP enzyme inhibitor in this fatal case [23]. Another hypothesis is that AM1220 had not been sufficiently metabolised before the death, leading to less metabolites with an incomplete metabolic pattern. The higher concentration of AM1220 in plasma than its potential *N*-dealkylated metabolite is in line with this hypothesis (hydroxy and dihydroxy metabolites were not quantified), as well as the detection of hydroxy and dihydroxy metabolites in plasma despite their absence in urine [10]. For these reasons, it would be ideal to compare the in vitro metabolism data with multiple in vivo data.

### Suggested markers

The three most abundant metabolites in HLM and fungus incubations were dihydrodiol (H3), demethylation (H7) and hydroxylation at the piperidine moiety (H8), and hydroxylation at the naphthalene moiety (F4), dihydrodiol (F1) and demethylation (F6), respectively. Based on the high abundance observed in vitro, these metabolites could be potential in vivo markers of AM1220 intake. The desmethyl metabolite and hydroxy metabolite at the piperidine moiety are particularly interesting as the same transformation pathways were predominant for AM1241, an analogue of AM1220 with the naphthalene moiety replaced by 2-iodo-5-nitrophenyl group, in HLM and rat microsomes incubations [32]. The dihydrodiol metabolites are also promising, as dihydrodiol and hydroxy metabolites were the most abundant metabolites of AM2201 in authentic human urine samples, when excluding oxidative defluorination [34]. Since AM1220 does not undergo oxidative defluorination, dihydrodiol formation may be an abundant in vivo metabolic pathway.

## Conclusions

A potent synthetic cannabinoid AM1220 was incubated in HLM and *C. elegans* to elucidate the structures of the in vitro metabolites. Metabolic stability of AM1220 was estimated from HLM incubation and the estimated in vitro half-life and hepatic extraction ratio indicated AM1220 to be a high clearance drug. LC–QTOF-MS analysis of HLM and *C. elegans* samples resulted in detection of a total of 11 metabolites (nine and seven metabolites in respective samples) and they consisted of hydroxy, dihydroxy, desmethyl, dihydrodiol, and dihydrodiol-desmethyl metabolites. The results did not match the in vivo metabolism previously reported; however it should be noted that the results in the study were based on a single postmortem sample. Based on the in vitro data, hydroxy, desmethyl and dihydrodiol metabolites are deemed suitable urinary markers of AM1220 intake. These data should help toxicological and clinical laboratories to identify AM1220 consumption from human urine samples.
